# Clinical Utility of Quantitative MRI Parameters for Differentiation of Renal Tumor Subtypes and Who Grades: A Multiparametric Approach with Internal Cortical Reference

**DOI:** 10.3390/jcm15103653

**Published:** 2026-05-09

**Authors:** Ekrem Anil Sari, Serap Sari, Canan Altay, Altug Didikoglu, Furkan Mert Kervan, Mustafa Secil

**Affiliations:** 1 Department of Radiology, Dokuz Eylul University, 35220 Izmir, Turkey; 2 Radiology Department, Ataturk Training and Research Hospital, 35360 Izmir, Turkey; 3 Department of Molecular Biology and Genetics, Izmir Institue of Technology, 35430 Izmir, Turkey

**Keywords:** renal tumor, magnetic resonance imaging, quantitative MRI, ADC, T2* mapping, R2*, renal cell carcinoma, tumor grading

## Abstract

**Background/Objectives:** To evaluate the clinical utility and diagnostic performance of quantitative MRI parameters (T1, T2*, R2*, and ADC) in differentiating renal tumor subtypes and WHO grades, and to assess their potential role in non-invasive tumor characterization. **Methods:** This retrospective study included 82 patients with histopathologically confirmed renal tumors who underwent preoperative contrast-enhanced MRI between July 2019 and January 2024. Quantitative measurements were obtained from tumor regions and contralateral healthy renal cortex using standardized ROI-based analysis. Parameters included T2*, native and post-contrast T1, R2* (1/T2*), and ADC values. Interobserver agreement was assessed. A Random Forest model was used as a supplementary analytical tool. **Results:** The cohort included 82 patients (mean age: 59.3 years). Tumors were classified into multiple subtypes, with clear cell carcinoma being the most common (n = 46). High-grade tumors (WHO grades 3–4) demonstrated significantly lower ADC values (*p* = 0.029) and larger tumor size (*p* = 0.0017). Significant differences in T2*, R2*, and ADC values were observed across tumor subtypes (*p* < 0.05). Quantitative MRI parameters demonstrated moderate discriminatory performance, with ADC emerging as the most robust biomarker. The Random Forest model achieved an overall accuracy of 93.2%, primarily driven by ADC and post-contrast T1 values. **Conclusions:** Quantitative MRI parameters, particularly ADC, provide clinically meaningful non-invasive biomarkers for renal tumor characterization. Their combined interpretation, supported by contralateral renal cortex comparison, may enhance clinical decision-making. Further validation in larger cohorts is warranted.

## 1. Introduction

Renal masses are increasingly detected incidentally with the widespread use of cross-sectional imaging, encompassing a heterogeneous spectrum of neoplasms with distinct histopathological subtypes, molecular profiles, and clinical behaviors [1]. Despite advances in imaging techniques, accurate non-invasive characterization of these lesions remains a significant clinical challenge, particularly in distinguishing tumor subtypes and predicting histopathological grade prior to treatment.

Tumor grade is a critical determinant of prognosis and therapeutic decision-making in renal cell carcinoma. High-grade tumors (WHO grades 3–4) are associated with substantially worse clinical outcomes compared to low-grade tumors (grades 1–2), with reported survival rates varying markedly between these groups [2]. Therefore, reliable preoperative estimation of tumor grade may facilitate risk stratification and guide individualized management strategies.

Current treatment approaches for renal tumors range from active surveillance to surgical resection, ablation therapies, and systemic treatments. In this context, magnetic resonance imaging (MRI) has emerged as a valuable non-invasive tool due to its superior soft-tissue contrast and its ability to provide both anatomical and functional information. Beyond conventional imaging, quantitative MRI techniques enable objective assessment of tissue characteristics and tumor microenvironment.

Quantitative parameters such as native and post-contrast T1 relaxation times, T2* relaxation times, apparent diffusion coefficient (ADC), and derived R2* values have shown promise as imaging biomarkers reflecting cellularity, vascularity, and tissue composition [3]. Among these, ADC has been extensively studied and is widely accepted as a surrogate marker of tumor cellularity. However, the additional value of other quantitative parameters, particularly T2* and R2* metrics, remains incompletely understood [4,5,6]. Recent studies have explored multiparametric MRI and quantitative approaches for renal tumor characterization, including T1 mapping and diffusion-based analyses [7,8].

In contrast to previous studies, our approach integrates multiple quantitative MRI parameters together with the use of the contralateral healthy renal cortex as an internal reference, which may enhance clinical interpretability.

Moreover, the majority of previous studies have evaluated these parameters in isolation, limiting their clinical applicability. A comprehensive multiparametric approach may provide a more robust and reproducible characterization of renal tumors. In addition, normalization strategies using internal references, such as the contralateral renal cortex, have been insufficiently explored despite their potential to reduce inter-individual variability and improve measurement reliability.

In this context, the present study aims to investigate the clinical utility of a multiparametric MRI approach for the differentiation of renal tumor subtypes and WHO grades. Unlike prior studies, we integrated multiple quantitative MRI parameters and applied normalization using the contralateral renal cortex as an internal reference to enhance reproducibility and real-world applicability. Furthermore, a Random Forest-based machine learning model was incorporated as a supportive analytical tool to evaluate the combined diagnostic performance of imaging parameters rather than to establish a standalone predictive model.

Specifically, this study sought to: (1) assess interobserver agreement of quantitative MRI measurements; (2) evaluate differences in imaging parameters between tumor grades; (3) investigate variations among renal tumor subtypes; and (4) explore the potential clinical utility of these parameters in tumor classification [9].

## 2. Materials and Methods

### 2.1. Ethical Considerations

This retrospective study was conducted following approval from the Dokuz Eylul University Faculty of Medicine Ethics Committee (Decision No: 2023/28-11) and adhered to the principles outlined in the Declaration of Helsinki. The requirement for informed consent was waived due to the retrospective design of the study.

### 2.2. Population

This study included adult patients with histopathologically confirmed renal tumors who underwent surgical treatment for solid renal masses at our institution between July 2019 and January 2024. A total of 204 patients were initially identified during this period.

Among these, patients who had undergone preoperative MRI using a standardized renal imaging protocol that included full renal parenchymal coverage were selected for further evaluation (n = 100).

Exclusion criteria were as follows: MRI examinations performed at external institutions, age under 18 years, presence of cystic renal tumors, and inadequate image quality due to significant artifacts (e.g., motion-related degradation) that could compromise quantitative analysis.

After applying these criteria, 82 patients were included in the final study cohort.

### 2.3. MRI Protocol

All MRI examinations were performed on a 1.5-T system (Philips Healthcare, Best, The Netherlands).

The imaging protocol included conventional anatomical sequences such as axial and coronal T2-weighted turbo spin-echo (TSE), fat-suppressed T2-weighted imaging (SPIR), dual-phase gradient-echo (GRE) T1-weighted imaging, and dynamic contrast-enhanced GRE T1-weighted imaging.

Diffusion-weighted imaging (DWI) was acquired using a b-value of 1000 s/mm^2^, and corresponding apparent diffusion coefficient (ADC) maps were automatically generated (TR/TE = 2084/101 ms; slice thickness = 5 mm).

T2* mapping and derived R2* values were obtained using an mDixon Quant sequence (TR = 5.32 ms; matrix = 192 × 192; slice thickness = 3 mm).

T1 mapping was performed both before and after contrast administration using identical acquisition parameters (TR = 2.14 ms; TE = 0.956 ms; slice thickness = 10 mm).

### 2.4. Image Analysis

Preoperative MRI datasets were evaluated using the institutional picture archiving and communication system (PACS) on a dedicated workstation (Sectra Workstation IDS7, Version 24.2.16.6066; Sectra AB, Linköping, Sweden). Demographic, clinical, and histopathological information was retrieved from the hospital database.

**Figure 1:**
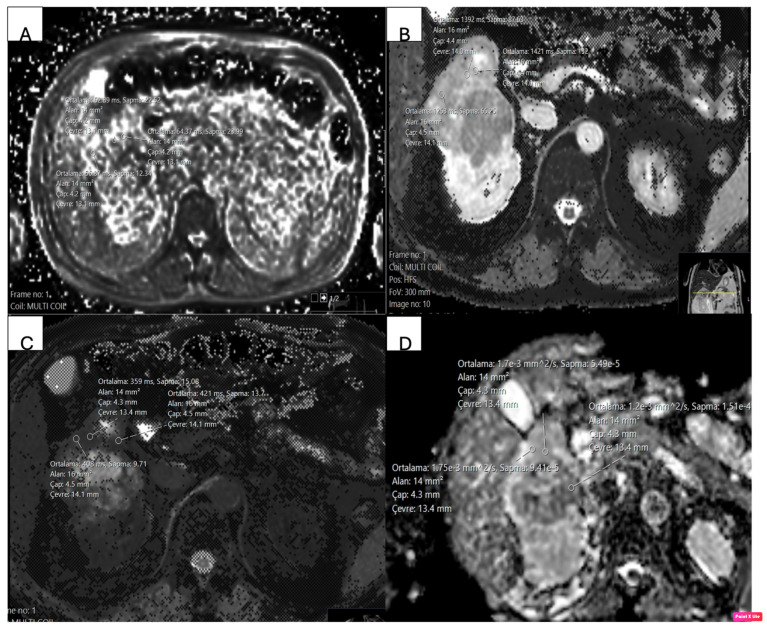
Quantitative MRI measurements obtained from renal tumor tissue. (**A**) T2* mDixon Quant image, (**B**) native T1 mapping, (**C**) post-contrast T1 mapping, and (**D**) ADC map. Regions of interest (ROIs) were placed within the solid components of the tumor, and mean values were calculated. R2* values were derived as the reciprocal of T2*.

Quantitative analysis was performed on the enhancing solid portions of the renal tumors. For each lesion, three circular regions of interest (ROIs) with an area ranging from 10 to 20 mm^2^ (mean approximately 15 mm^2^) were placed within representative tumor regions, and the average value was used for statistical analysis. Areas demonstrating hemorrhage, necrosis, cystic degeneration, or macroscopic fat were systematically excluded. In addition, regions exhibiting marked signal heterogeneity or high variability were avoided to ensure measurement consistency [Figure 1]. ROIs were deliberately kept small to minimize partial volume effects and to avoid inclusion of non-tumoral components such as necrotic, cystic, or hemorrhagic areas. Placement was performed subjectively on the most solid-appearing tumor regions on representative slices.

Tumor T2*, native T1, and post-contrast T1 relaxation times were recorded in milliseconds (ms), and R2* values were derived as the reciprocal of T2* (1/T2*). Apparent diffusion coefficient (ADC) values were obtained from ADC maps and expressed in mm^2^/s. ADC values were not normalized.

**Figure 2:**
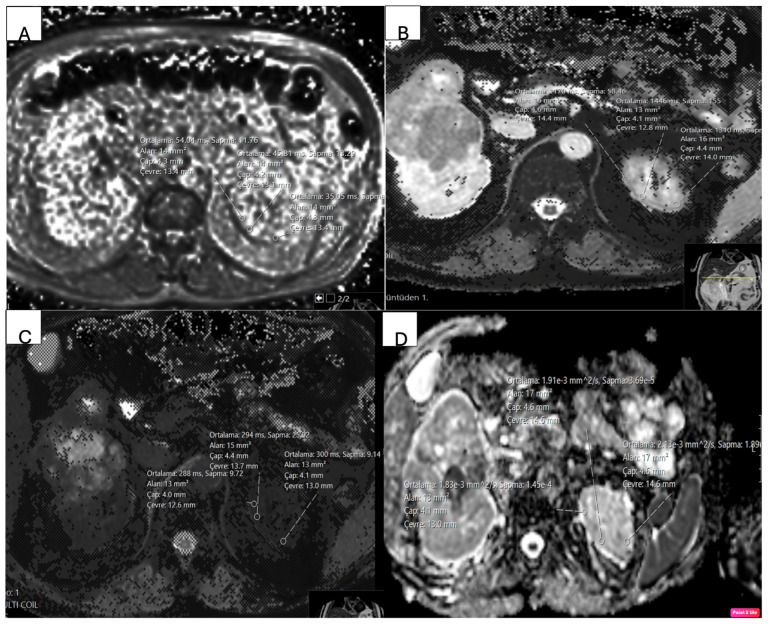
Quantitative MRI measurements obtained from the contralateral healthy renal cortex. (**A**) T2* mDixon Quant image, (**B**) native T1 mapping, (**C**) post-contrast T1 mapping, and (**D**) ADC map. Regions of interest (ROIs) were placed within the renal cortex, avoiding collecting systems and cystic structures, and mean values were calculated. R2* values were derived as the reciprocal of T2*.

To account for inter-individual variability, corresponding measurements were also obtained from the cortex of the contralateral normal kidney in each patient, carefully avoiding the collecting system and simple cysts [Figure 2].

All measurements were initially performed by a radiologist with 5 years of experience in abdominal imaging and subsequently repeated by a second independent radiologist with 8 years of experience, who was blinded to the initial measurements, in order to assess interobserver reproducibility. Standardized ROI placement was applied across all cases to minimize observer-dependent variability.

### 2.5. Statistical Analysis

Descriptive statistics were reported as frequencies for categorical variables and as mean ± standard deviation or median with interquartile range (IQR) for continuous variables, depending on data distribution. Normality was assessed using visual inspection of histograms in combination with the Shapiro–Wilk test. As the MRI-derived variables did not follow a normal distribution, non-parametric statistical methods were applied throughout the analysis.

Interobserver agreement was evaluated using Spearman’s rank correlation coefficient. For grading analysis, tumors were grouped into low-grade (WHO grades 1–2) and high-grade (WHO grades 3–4) categories. In addition, the relationship between tumor size (maximum diameter in mm) and WHO grade was investigated.

For subtype evaluation, the most frequently encountered tumor types (clear cell, papillary, and chromophobe renal cell carcinoma) were analyzed using a binary classification framework (presence vs. absence).

Comparisons between independent groups were performed using the Mann–Whitney U test, while paired comparisons between tumor tissue and the contralateral normal renal cortex were conducted using the Wilcoxon signed-rank test. A two-tailed *p*-value of <0.05 was considered statistically significant. Statistical analyses were performed using IBM SPSS Statistics for Windows, Version 29.0 (IBM Corp., Armonk, NY, USA).

### 2.6. Machine Learning

A Random Forest algorithm was used as a supplementary analytical tool to differentiate renal tumor tissue from healthy renal cortex. Model parameters were optimized using out-of-bag (OOB) error estimation and 10-fold cross-validation, with the number of variables randomly sampled at each split (mtry) set to 5. To reduce overfitting, the node size was set to 10 and the maximum number of nodes was limited to 5.

The dataset from the first observer was used for training, while the second observer dataset was used as independent test data. All statistical analyses were performed using R software version 4.3.2 (R Foundation for Statistical Computing, Vienna, Austria), and data visualizations were generated using the ggplot2 package.

A Random Forest algorithm was employed as a supplementary analytical approach to support the differentiation between renal tumor tissue and the contralateral normal renal cortex. Model performance was optimized using out-of-bag (OOB) error estimation in conjunction with 10-fold cross-validation. The number of variables randomly selected at each split (mtry) was set to 5. To mitigate overfitting, the minimum node size was set to 10, and the maximum number of terminal nodes was restricted to 5.

For model development, measurements obtained by the first observer were used as the training dataset, while those obtained by the second observer served as an independent test set. This approach allowed for an additional assessment of model robustness and reproducibility.

All statistical and machine learning analyses were performed using R software, and graphical visualizations were generated using the ggplot2 package.

## 3. Results

### 3.1. Patient Characteristics and Tumor Subtypes

The study cohort comprised 82 patients, including 32 women and 50 men, with a mean age of 59.3 ± 13.7 years (range: 20–79 years). The mean tumor size was 60.7 ± 34.2 mm, with values ranging from 16 to 170 mm.

**Figure 3:**
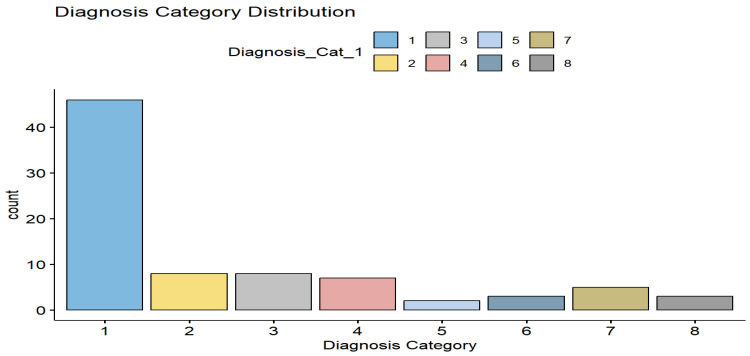
Distribution of renal tumor subtypes according to diagnostic categories. The *y*-axis represents the number of patients, and the *x*-axis represents diagnostic categories. Subtypes were defined as follows: 1 = clear cell renal cell carcinoma (ccRCC), 2 = papillary renal cell carcinoma (pRCC), 3 = chromophobe renal cell carcinoma (chRCC), 4 = oncocytoma, 5 = unclassified renal cell carcinoma and sarcomatoid clear cell renal cell carcinoma, 6 = squamous cell carcinoma and high-grade papillary urothelial carcinoma, 7 = fat-containing tumors including angiomyolipoma (AML) and well-differentiated liposarcoma, and 8 = lymphoma. The number of patients in each group was as follows: 1 = 46, 2 = 8, 3 = 8, 4 = 7, 5 = 2, 6 = 3, 7 = 5, and 8 = 3.

WHO grading information was available for 52 patients, primarily those with clear cell and papillary renal cell carcinoma. Among these, 18 tumors were classified as high-grade (grades 3–4), while 34 were categorized as low-grade (grades 1–2). The distribution of renal tumor subtypes is illustrated in Figure 3, with clear cell renal cell carcinoma representing the most prevalent histological subtype.

### 3.2. Interobserver Agreement

Interobserver agreement analysis showed strong agreement across all quantitative MRI parameters obtained from renal tumor measurements (Figure S1). The highest level of agreement was observed for post-contrast T1 values (R = 0.97), whereas the lowest correlation was noted for R2* values (R = 0.87). Despite this variability, all parameters were found to be statistically significant (*p* < 0.0001), indicating overall high reproducibility.

For measurements derived from the contralateral normal renal cortex, correlations ranged from moderate to strong (Figure S2). The highest agreement was found for T2* mDixon Quant values (R = 0.94), while ADC values demonstrated comparatively lower correlation (R = 0.53). Although all correlations remained statistically significant (*p* < 0.0001), the degree of agreement was generally lower than that observed in tumor measurements.

### 3.3. Comparison of MRI Data Between WHO Grades

#### 3.3.1. Comparison of WHO Grades (Severe and Non-Severe) with MRI Data

**Figure 4:**
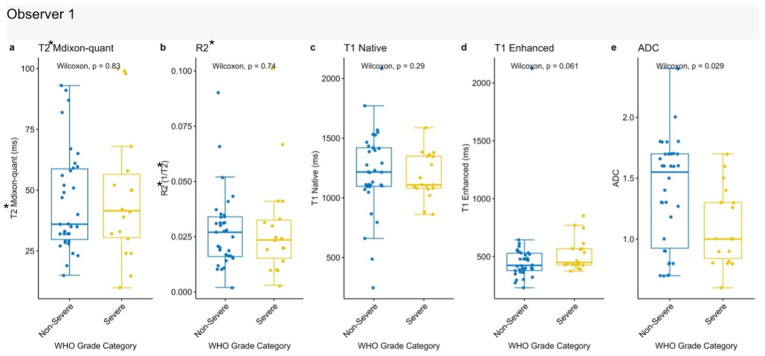
Comparison of quantitative MRI parameters according to WHO tumor grades. Box plots demonstrate differences between low-grade (grades 1–2) and high-grade (grades 3–4) tumors. (**a**) T2* mDixon Quant, (**b**) R2*, (**c**) native T1, (**d**) post-contrast T1, and (**e**) ADC values are shown. Statistical comparisons were performed using the Wilcoxon signed-rank test.

ADC values showed a significant difference between low-grade and high-grade tumors (*p* = 0.029), with lower values observed in high-grade lesions. No meaningful differences were detected for the remaining quantitative MRI parameters between the two groups [Figure 4].

#### 3.3.2. Comparison of Tumor Size with WHO Grades

**Figure 5:**
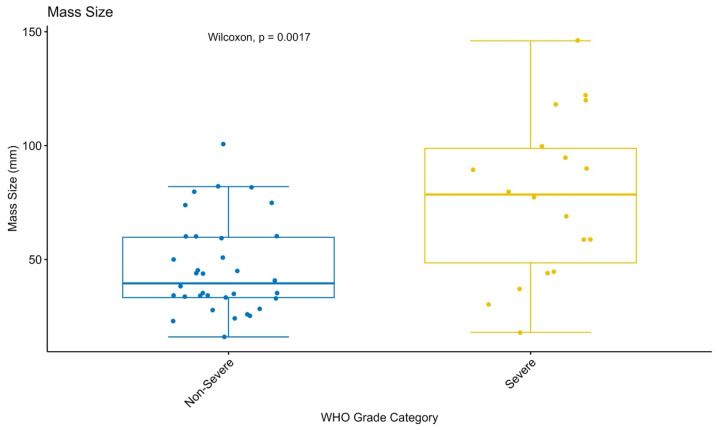
Comparison of tumor size according to WHO tumor grades. Box plots demonstrate differences in maximum tumor diameter (mm) between low-grade (grades 1–2) and high-grade (grades 3–4) tumors. Statistical comparisons were performed using the Wilcoxon signed-rank test.

Tumor size was significantly higher in high-grade tumors compared to low-grade tumors (*p* = 0.0017) [Figure 5], indicating a potential association between tumor size and histopathological aggressiveness.

### 3.4. Demonstration of Differences Between Diagnostic Categories

#### 3.4.1. Clear Renal Cell Carcinoma

**Figure 6:**
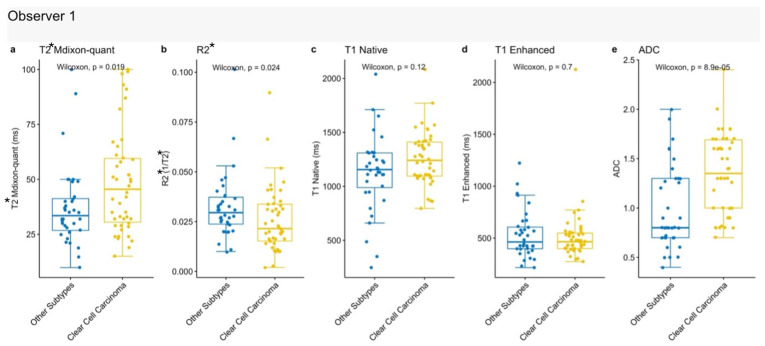
Comparison of quantitative MRI parameters between clear cell renal cell carcinoma and other renal tumors. (**a**) T2* mDixon-quant; (**b**) R2*; (**c**) Native T1; (**d**) Enhanced T1; (**e**) ADC values.

T2* mDixon Quant, R2*, and ADC values differed significantly between clear cell renal cell carcinoma (ccRCC) and other tumor subtypes. The *p*-values for T2* mDixon Quant and R2* were 0.019 and 0.024, respectively, while ADC demonstrated the strongest statistical significance (*p* < 0.001). Compared to other tumor types, ccRCC exhibited higher T2* mDixon Quant and ADC values, and lower R2* values [Figure 6], suggesting a distinct quantitative MRI profile for this subtype.

#### 3.4.2. Papillary Renal Cell Carcinoma

**Figure 7:**
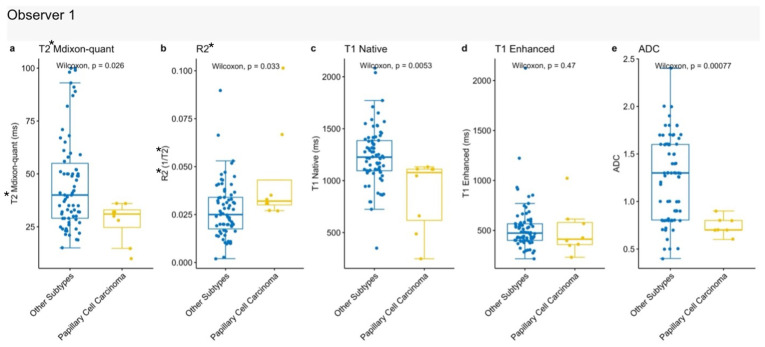
Comparison of quantitative MRI parameters between papillary renal cell carcinoma and other renal tumors. (**a**) T2* mDixon-quant; (**b**) R2*; (**c**) Native T1; (**d**) Enhanced T1; (**e**) ADC values.

When papillary renal cell carcinoma (pRCC) was compared with other tumor subtypes, all quantitative MRI parameters showed statistically significant differences except for post-contrast T1 values. The most pronounced differences were observed for ADC (*p* = 0.00077) and native T1 (*p* = 0.0053). T2* mDixon Quant and R2* values also demonstrated statistically significant, albeit less marked, differences (*p* = 0.026 and *p* = 0.033, respectively). Compared to other tumor types, pRCC demonstrated a pattern of lower T2* mDixon Quant, native T1, and ADC values, and higher R2* values [Figure 7], suggesting a distinct and potentially identifiable quantitative MRI signature.

#### 3.4.3. Chromophobe Renal Cell Carcinoma

**Figure 8:**
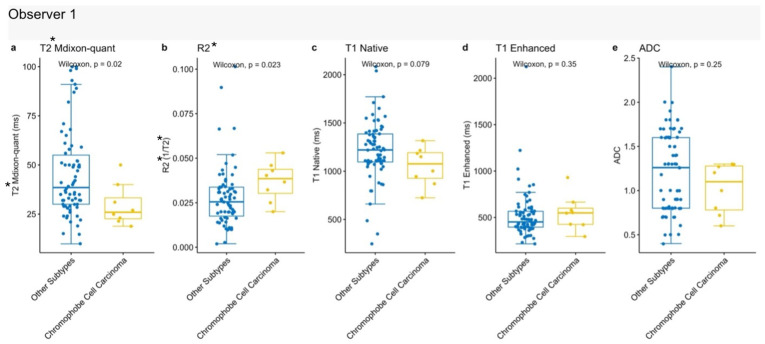
Comparison of quantitative MRI parameters between chromophobe renal cell carcinoma and other renal tumors. (**a**) T2* mDixon-quant; (**b**) R2*; (**c**) Native T1; (**d**) Enhanced T1; (**e**) ADC values.

In comparisons between chromophobe renal cell carcinoma (chRCC) and other tumor subtypes, T2* mDixon Quant and R2* values differed significantly, whereas no significant differences were observed for the remaining parameters. Compared to other tumor types, chRCC was associated with higher T2* mDixon Quant and R2* values [Figure 8].

### 3.5. Comparison of Tumor Tissue and Contrasting Healthy Kidney Cortex According to MRI Measurement Values in All Patients

**Figure 9:**
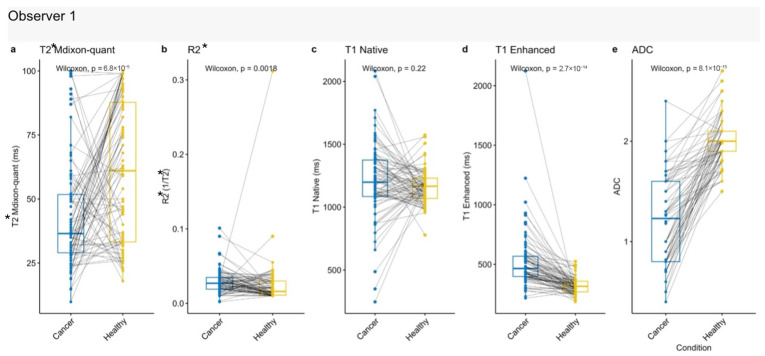
Paired comparison of quantitative MRI parameters between renal tumors and contralateral healthy renal cortex. Box plots demonstrate differences between tumor tissue and healthy cortex across all patients. (**a**) T2* mDixon Quant, (**b**) R2*, (**c**) native T1, (**d**) post-contrast T1, and (**e**) ADC values are shown. Paired comparisons were performed using the Wilcoxon signed-rank test.

In the cohort of 82 patients, quantitative MRI parameters of renal tumors were compared with those of the contralateral normal renal cortex using paired analysis. Significant differences were observed for T2* mDixon Quant, R2*, post-contrast T1, and ADC values, whereas native T1 did not demonstrate a significant difference (*p* = 0.22). Among the significant parameters, ADC showed the strongest statistical significance (*p* < 0.0001), while R2* exhibited a comparatively weaker, although still significant, association (*p* = 0.0018). Compared to the contralateral normal cortex, renal tumors demonstrated lower T2* mDixon Quant and ADC values, and higher R2* and post-contrast T1 values [Figure 9], highlighting the potential of these parameters for tumor detection and tissue differentiation.

### 3.6. Machine Learning Analysis

A Random Forest model was applied as a supplementary analytical approach to differentiate renal tumor tissue from the contralateral normal renal cortex using quantitative MRI parameters. The dataset obtained from the first observer was used for model training, while the dataset from the second observer served as an independent test set.

Model performance reached a plateau before 500 trees; however, 500 trees were retained for consistency across analyses. In addition, 10-fold cross-validation was performed within the training dataset to improve model robustness and reduce the risk of overfitting.

**Figure 10:**
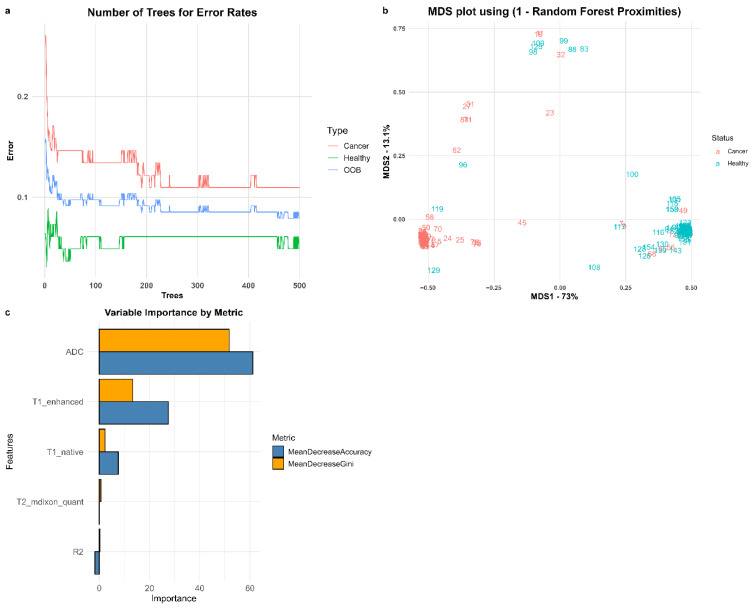
Random Forest model analysis. (**a**) Relationship between the number of trees and error rates; (**b**) Multidimensional scaling (MDS) plot based on Random Forest proximities; (**c**) Variable importance plot showing mean decrease in accuracy and mean decrease in Gini index for each parameter.

When all five parameters were included, the model demonstrated clear separation between tumor and non-tumor samples [Figure 10]. The model achieved an overall accuracy of 93.2% on the independent test dataset. Detailed classification performance and error rates are provided in Table 1.

Feature importance analysis indicated that ADC and post-contrast T1 values were the most influential variables, demonstrating the highest mean decrease in accuracy and Gini index [Figure 10]. Threshold analysis further suggested that these parameters may contribute to effective discrimination between tumor and healthy tissue, consistent with the findings presented in Figure 9.

However, these findings should be interpreted with caution, as both the training and test datasets were derived from the same patient cohort, albeit evaluated by different observers, and external validation was not performed.

## 4. Discussion

MRI is widely used for the diagnosis and staging of renal tumors. In addition to conventional sequences, advanced techniques such as T1, T2, T2* mapping, and diffusion-weighted imaging (DWI) contribute to tumor characterization. Although these techniques are more commonly applied in cardiac imaging, they have also been investigated in brain tumors, lymph nodes, and renal tumors [10,11]. However, studies evaluating T1 mapping, post-contrast T1, T2*, R2*, and ADC values in renal tumors remain limited [10,11,12,13,14,15,16]. The present study integrates multiple quantitative MRI parameters, including native and post-contrast T1, T2* mapping, R2*, and ADC values, within a single analysis. In addition, measurements from the contralateral healthy renal cortex were used as an internal reference, which may enhance the clinical interpretability of quantitative imaging.

Regarding the measurement methodology, quantitative values were obtained from both tumor tissue and the contralateral renal cortex using standardized ROI placement. Interobserver agreement analysis showed strong correlations, particularly for post-contrast T1 values (R = 0.97), indicating good reproducibility. In contrast, ADC measurements in the healthy cortex demonstrated moderate agreement, likely due to the absence of a clearly defined target region and increased variability in ROI placement.

Among all parameters, ADC showed the most consistent association with tumor grade. In line with previous findings by Mytsyk et al., ADC values decreased with increasing tumor grade [14]. Similarly, ADC values were significantly lower in high-grade tumors compared to low-grade tumors in our cohort. This finding may reflect increased cellularity and reduced extracellular space in higher-grade tumors, resulting in greater diffusion restriction.

Previous studies have also explored the relationship between MRI parameters and tumor grade. Adams et al. reported higher native T1 values in higher-grade tumors, attributed to increased collagen content [11]. They also observed a trend toward larger tumor size in higher-grade tumors. In another study, T2 mapping values were found to be longer in low-grade tumors [15]. In contrast, our study did not demonstrate statistically significant differences in native T1 or T2* values between different grade groups. These discrepancies may be related to differences in tumor composition, inclusion of multiple tumor subtypes, and methodological variations between studies.

Consistent with prior literature, tumor size was significantly associated with tumor grade in our cohort [17]. However, with the increasing detection of incidental renal tumors, tumor size alone may not be a reliable indicator of tumor aggressiveness.

In subtype analysis, clear cell, papillary, and chromophobe renal cell carcinomas were the most common tumor types. Quantitative MRI parameters demonstrated significant differences between subtypes. Clear cell tumors showed higher T2* and ADC values and lower R2* values compared to other subtypes. Papillary tumors demonstrated lower T2* and ADC values and higher R2* values, consistent with previous reports showing lower T2 signal intensity in papillary tumors [18]. Similarly, ADC values were significantly lower in papillary tumors, in agreement with the findings of Çolakoğlu et al. [19]. Chromophobe tumors demonstrated distinct differences in T2* and R2* values, although comparable data in the literature remain limited.

When comparing tumor tissue with the contralateral healthy renal cortex, all parameters except native T1 showed significant differences. These findings suggest that quantitative MRI parameters, particularly ADC, may provide useful non-invasive biomarkers for tumor detection [6].

A Random Forest model was applied as a supplementary analytical tool, achieving an overall accuracy of 93.2% in differentiating tumor tissue from healthy cortex. However, potential overfitting cannot be excluded, as the training and test datasets were derived from the same patient cohort. In line with previous reviews, the application of artificial intelligence in radiology requires standardized datasets and robust validation for clinical implementation [20].

This study has several limitations. First, the relatively small sample size and uneven distribution of tumor subtypes may limit generalizability. Second, the lack of prior studies using a similar multiparametric approach restricts direct comparison with existing literature. Third, the post-contrast T1 sequence was acquired in a single phase, which may have limited the evaluation of dynamic contrast behavior. Fourth, the uneven distribution of tumor subtypes, with relatively small numbers of papillary and chromophobe tumors, which may limit statistical power and generalizability. In addition, although statistically significant differences were identified, clinically applicable decision thresholds were not established. Threshold-based analyses (e.g., sensitivity and specificity using optimal cutoffs such as the Youden index) were not performed due to the limited sample size and class imbalance. Future studies with larger and more balanced cohorts are needed to enable reliable threshold determination and clinical implementation. Finally, ROI placement was performed manually, introducing potential observer variability compared to automated methods.

Despite these limitations, the findings suggest that multiparametric quantitative MRI, particularly ADC, may provide clinically meaningful information for renal tumor characterization. Further studies with larger cohorts and external validation are warranted.

## 5. Conclusions

Quantitative MRI plays an important role in the non-invasive evaluation of renal tumors, providing valuable information on tumor subtype and grade through parameters such as T1, T2*, R2*, and ADC.

In this study, ADC values showed a significant association with tumor grade and tumor size. Multiparametric analysis further demonstrated that T2*, R2*, and ADC values in clear cell tumors; T2*, R2*, native T1, and ADC values in papillary tumors; and T2* and R2* values in chromophobe tumors may contribute to subtype differentiation.

When tumor measurements were compared with the contralateral normal renal cortex, all parameters except native T1 demonstrated significant differences. These findings suggest that quantitative MRI parameters, particularly ADC, may serve as clinically meaningful non-invasive biomarkers for renal tumor characterization.

A Random Forest model demonstrated high accuracy in differentiating tumor tissue from healthy cortex; however, these results should be interpreted with caution, and further validation in larger and independent cohorts is required.

Overall, quantitative MRI shows promise as a non-invasive tool for renal tumor characterization. Its integration into clinical practice may support more individualized decision-making, although standardization of imaging protocols and validation in larger, more homogeneous populations remain necessary.

## Figures and Tables

**Table 1:** Classification performance of the Random Forest model on the independent test dataset.

	Health Condition	Cancer	Healthy	Error Rate	Overall Accuracy
Test Data	Cancer	74	8	0.098	0.932
	Healthy	3	79	0.037	

## Data Availability

The data presented in this study are available on reasonable request from the corresponding author.

## Supplementary Materials

The following supporting information can be downloaded at: https://www.mdpi.com/article/10.3390/jcm15103653/s1, Figure S1: Interobserver agreement of kidney tumor measurements: 1st observer’s measurements are shown on the x-axis, 2nd observer’s measurements are shown on the y-axis. T2 mDixon (a), R2* (b), T1 native (c), T1 enhanced (d) and ADC (e) values for kidney tumors were compared to assess the agreement between the 1st and 2nd observer; Figure S2: Interobserver agreement of opposite kidney renal cortex measurements: 1st observer’s measurements are shown on the x-axis, 2nd observer’s measurements are shown on the y-axis. T2 mDixon (a), R2* (b), T1 native (c), T1 enhanced (d) and ADC (e) values of the opposite kidney healthy cortex measurements were compared to evaluate the agreement between the 1st and 2nd observer.
