# Supplementary material for: Clinical Utility of Quantitative MRI Parameters for Differentiation of Renal Tumor Subtypes and Who Grades: A Multiparametric Approach with Internal Cortical Reference
Source: J Clin Med. 2026 May 9;15(10):3653. doi: 10.3390/jcm15103653 (PMC13207497; doi:10.3390/jcm15103653)
Supplement: Supplementary file 1 [file jcm-15-03653-s001.zip › jcm-4243598-supplementary.pdf]

Supplementary Material for Review

Cancer Kidney

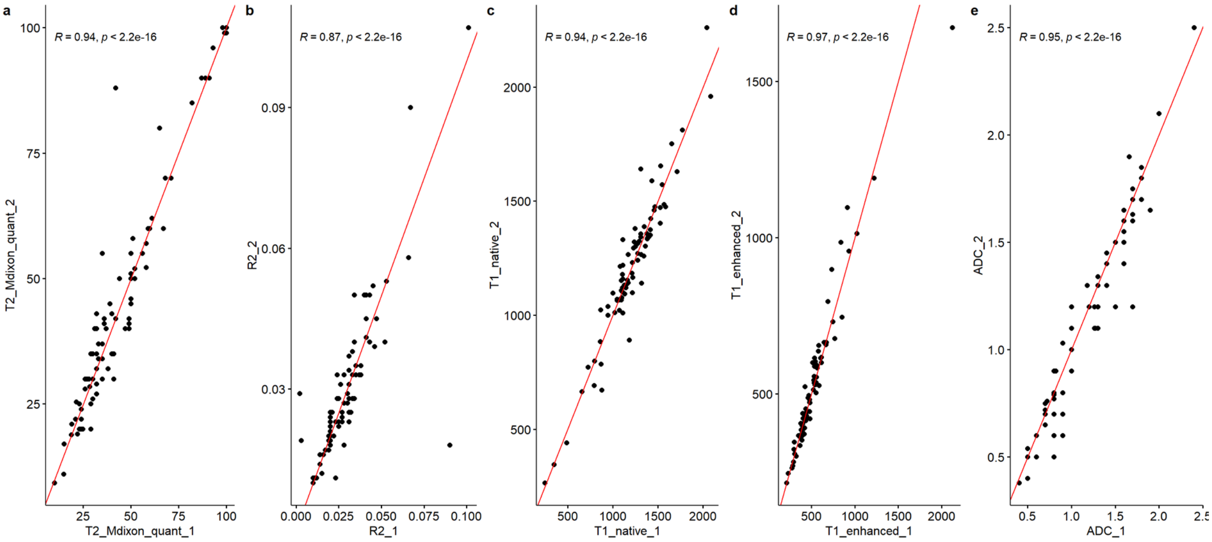

Figure S1 -Interobserver agreement of kidney tumor measurements: 1st observer's measurements are shown on the x-axis, 2nd observer's measurements are shown on the y-axis. T2 mDixon(a), R2*(b), T1 native(c), T1 enhanced(d) and ADC(e) values for kidney tumors were compared to assess the agreement between the 1st and 2nd observer.

Healthy Kidney

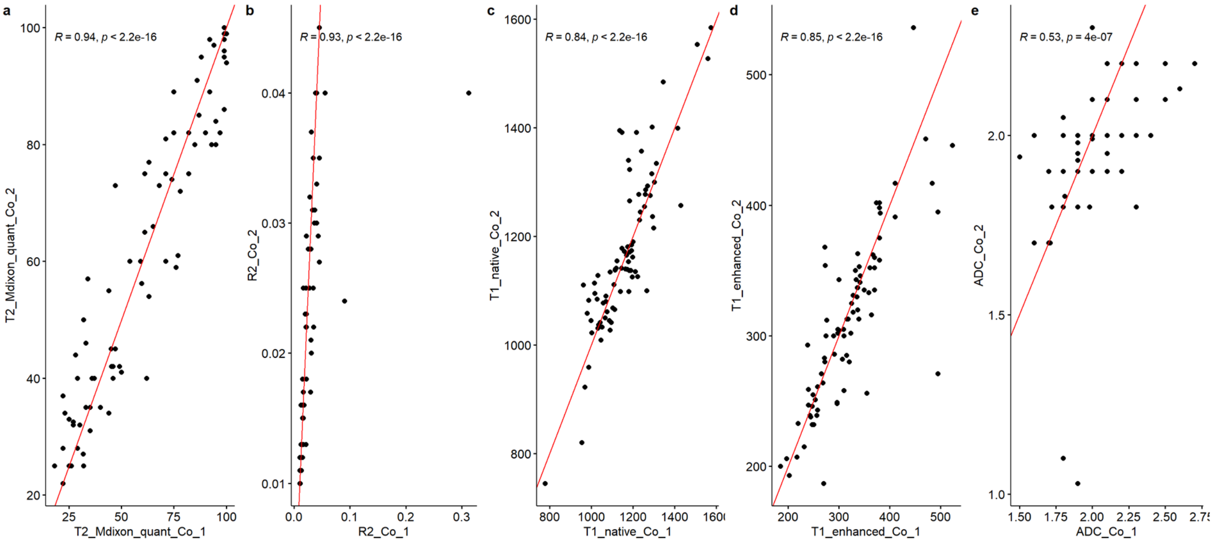

Figure S2 - Interobserver agreement of opposite kidney renal cortex measurements: 1st observer's measurements are shown on the x-axis, 2nd observer's measurements are shown on the y-axis. T2 mDixon(a), R2*(b), T1 native(c), T1 enhanced(d) and ADC(e) values of the opposite kidney healthy cortex measurements were compared to evaluate the agreement between the 1st and 2nd observer.
